# Complexities of health and acceptance of electronic health records for the Austrian elderly population

**DOI:** 10.1007/s10198-022-01451-z

**Published:** 2022-03-14

**Authors:** Nicole Halmdienst, Gerald J. Pruckner, Rudolf Winter-Ebmer

**Affiliations:** 1 Johannes Kepler University of Linz, Department of Economics, Altenberger Straße 69, 4040 Linz, Austria; 2 Christian Doppler Laboratory Aging, Health and the Labor Market, Linz, Austria; 3 Linz Institute of Technology, Linz, Austria; 4 Institute for Advanced Studies, Vienna, Austria

**Keywords:** Electronic health records, Health status, Social connectedness, SHARE survey, I11, I12, I18

## Abstract

We examine the personal health situation and how the complexities thereof affect the elderly Austrians’ willingness to accept electronic health records (EHR). Using data from the sixth wave of the SHARE survey in Austria, we find the complexity of individual health problems and the social integration of individuals influencing the acceptance of EHR. The higher the degree of multimorbidity, the more medication is prescribed, and the higher the number of hospital admissions, the higher is the acceptance of EHR. Having a chronical illness has a positive effect on EHR acceptance, whereas a pessimistic attitude and lack of joy in life, as indicators of depressive mood, have a negative impact. The results are mainly driven by women and younger patients aged between 50 and 70. People with poor social connection express lower acceptance of EHR.

## Introduction

The provision of medical services and prescription of medication in most health care systems involve numerous stakeholders, such as patients, physicians, clinics, and pharmacies. As the services are offered at different times and places, the necessary information for the adequate treatment of a patient is very often not available. To address this issue, several countries have developed Internet-based tools that can easily access and coordinate health information and make it available to patients and health care service providers. These electronic health records (EHR) typically include e-medication and electronic medical certificates to adequately improve the prescription of medical drugs, reduce the negative consequences of polypharmacy, and avoid unnecessary double and multiple medical examinations.

Although records contribute to patient safety by providing important information to the attending doctors and medication-dispensing pharmacists, the EHR acceptance rates of patients and physicians are relatively low [28]. In addition to the technical aspects of usability and interoperability for clinics, doctors, and pharmacies, privacy issues and trust are crucial factors for acceptance. Concerns in these areas, particularly pertaining to the misuse of sensitive data, are the main arguments against the broad use of EHR.

In this study, we examine whether and to what extent individual patient characteristics affect the acceptance of EHR. The individual’s physical state, mental health, and number of appointments with the health care system are potential determinants of the patient’s attitude toward the electronic provisioning of personalized health records. We examine whether patients who depend more on the availability of health data at all times (e.g., chronically ill persons) or those who require more diverse appointments with the health system (e.g., those with multimorbidity) have a more positive attitude toward EHR. A second important question relates to the role of social connectedness in the acceptance and adoption of EHR. We use data from the sixth wave of the Survey of Health, Ageing, and Retirement in Europe (SHARE) conducted in Austria. The survey respondents are asked questions on their attitude toward and use of new technologies, in particular related to ELGA (in German “Elektronische Gesundheitsakte”), the Austrian EHR system.

We find that multimorbidity, the number of medication prescriptions, and hospitalizations have a positive effect on a patient’s acceptance of EHR. Having a chronic illness also has a positive effect on EHR acceptance, whereas having a pessimistic attitude and lack of joy in life, indicating depressive moods, have a negative impact. These results are mainly observed among female patients and the younger patients aged between 50 and 70. People with poor social connections express lower acceptance of EHR.

*Literature:*  Non-technical studies in the literature have examined the acceptance of EHR and its determinants. The majority of these studies deal with cases, where health care providers are ready to adopt electronic information systems. Review papers have reported a multitude of barriers to physicians adopting EHR. The most frequently mentioned barriers are privacy and security concerns, high start-up cost, workflow changes, system complexity, lack of reliability, and interoperability [9]. In a recent multi-center survey study in Germany, Ploner et al. [28] show that the trust of doctors (and patients) in health care providers exceeds their trust in other institutions, such as private firms. Therefore, the authors argue that the health care providers should offer the personal health record infrastructure to their patients. Besides the trust in the institution offering the EHR and privacy concerns, social influence and previous experience with health IT play a key role in EHR acceptance. Steininger and Stiglbauer [30] conduct a nationwide survey of Austrian general practitioners and medical specialists in private practice. The survey results show that apart from the data protection it provides, public debates on the topic and the extent of previously used health IT functions determine the physicians’ perceived usefulness of the program. Hackl et al. [16] confirm these results in their survey study on the acceptance of the Austrian e-medication system. They show that doctors would have a positive feeling about it if the software vendor could provide sufficient support.

Systematic literature reviews identify the variables promoting the patients’ acceptance of consumer health information technology. Tao et al. [31] report that the current literature largely focuses on user characteristics related to health and health care, IT experience, or personality. In their review, Or and Karsh [27] do not find consistency in the influence of patient characteristics on consumer health information technology. The majority of studies find a significantly negative impact of age, while one-third of the papers considered report insignificant estimates of age impact. Gender demonstrates no effect in most cases, while more than two-thirds of the papers considered find the acceptance of consumer health information technology increasing with patient education. Moreover, an increase in acceptance is associated with prior experience and computer health technology use. Very few studies examine the impact of health status variables; they present mixed results. While some papers find a positive association between better health and increased acceptance [8], others report a negative correlation [20, 24]. Very little evidence exists on the predictive power of social factors, such as subjective norms, perceived social pressure, or social participation.

## Institutional background—ELGA

Numerous countries worldwide have implemented and developed EHR systems, with a wide variation in use rates. At the European level, Denmark, Finland and Sweden are considered to be the forerunners [3]. Together with Norway and Scotland, these countries have always recorded the highest rates in the use of EHR systems by primary care physicians, medical specialists, and hospitals [25]. In their recent study on cross-institutional availability of patient data for health care professionals and patients, Ammenwerth et al. [1] find that only very few countries guarantee full access to health record data and allow health care providers and patients to fully add relevant data. The study delivers the best outcome of all eHealth indicators in Europe for Finland, followed by Sweden. Austria was able to improve on individual indicators between 2017 and 2019.

ELGA is the Austrian EHR system. It provides a Web-based infrastructure for patients, hospitals, physicians, care facilities, and pharmacies to access individual health data and work together more efficiently with the treatment chain [11]. The information available covers, among others, medication, medical examination, blood group, and radiology and laboratory reports. The data are provided in a structured form to patients and health care providers, allowing for complete traceability of the patient’s medical history. This is intended to promote the quality of care and patient safety, and avoid multiple examinations [2].

The legal basis for establishing the program is the ELGA Act (in German “Elektronische-Gesundheitsakte-Gesetz”) promulgated in January 2013. This Act states the rights and obligations of health care providers and regulates the protection and security of data. It has been gradually introduced to the health care providers since December 2015; its full technical implementation in nursing homes and home care providers took place in 2021. ELGA allows patients to restrict their health information or fully opt out of the program. Approximately 3.4 % of patients opted out of the program as of February 2020 [13]. Finally, the system provides information as to who has accessed the individual’s data and at what time [2].

*The negative role of physician representatives:* The professional representation of physicians has taken a very critical stance towards ELGA over a long period of time. In January 2014, the entire leadership of the Austrian Association of General Practitioners (in German “Österreichischer Hausärzteverband”) submitted its withdrawal from ELGA. At the same time, an organized advertising campaign in doctors’ offices urged patients to also opt out of the EHR system. The alleged lack of data protection was the central argument of the medical profession against ELGA. The Austrian Medical Association also maintained its strong ELGA criticism until after its introduction in 2015, citing an unacceptably high level of bureaucracy and questionable benefits for patients and doctors.^1^

ELGA was scientifically evaluated in 2019 [7]. The evaluation report showed that 75 % of the medical records were captured in a structured form by 2019, with steady rise in use of the system. A survey of doctors in resident practices conducted during the course of the evaluation showed that 64 % of doctors perceived the program as a concrete benefit, whereas another 60 % found it too time-intensive [10]. The evaluation established five measures for further advancement: improve the completeness of ELGA, improve the usability of the system, improve the technical quality of medical records, reduce the time required for usage, and enhance the trust in ELGA.

Two recent reports complement the existing evaluation studies. A telephone survey of 124 service providers (physicians, hospitals, and pharmacies) conducted between June and July 2021 revealed that 63 % of respondents had a good or very good impression of ELGA [14]. The overall impression increased as the digital affinity of physicians and health care facilities went up. 6 out of 10 service providers used the e-vaccination and e-medication applications, while electronic medical diagnoses were only used by around 40 %. Among those who did not use ELGA, non-contracted physicians were disproportionately represented. Overall, the previously mentioned resistance of the medical profession to ELGA has obviously decreased.

A predominantly web-based representative survey of the Austrian population between 16 and 75 years showed that 71 % of respondents were familiar with ELGA and 50 % rated ELGA as personally useful [12]. In the sample, which included around 1300 people, the availability of important medical data in emergencies was cited as the greatest strength. Conversely, the lack of influence over what happened to the data was seen as the greatest weakness of EHR. The survey also showed that only a quarter of all respondents felt well informed about ELGA.

## Data and sample

Our analysis uses data from the 6th wave of SHARE in Austria, data release 6.0.0 [4]. SHARE is a pan-European multidisciplinary panel survey that collects micro data on health, socio-economic status, and social networks. The SHARE database currently provides data of more than 120,000 individuals aged 50 years or older in 27 European countries and Israel. The 6th SHARE data collection took place from January to September 2015. In the sixth wave in Austria, 3,402 individual panel interviews and 159 end-of-life interviews of the surviving dependents of deceased panel respondents were conducted. The individual response rate was 82 % [5]. In addition to the harmonized computer-assisted interviews, a country-specific paper-and-pencil questionnaire was distributed to the panel respondents with questions about their attitude toward new technologies. The response rate of the paper-and-pencil questionnaire was 91 %, representing 3,103 Austrian respondents aged 50 years or older.

As we use panel data, we need to take into account the sampling errors, non-response, and panel attrition. Moreover, the youngest cohorts in the Austrian data are underrepresented, because the last refreshment sample in SHARE wave 4 was drawn in 2011. Therefore, we weight the data using calibrated individual weights from the 6th wave of SHARE [5]. We note whether the results are weighted or not in each table. Finally, we drop 101 observations that have not answered the EHR question. The final sample consists of 2984 observations.

### Familiarity with survey subject

The interview period (from January to September 2015) consisted of the months immediately before the EHR system was gradually introduced to the Austrian health care sector. To correctly interpret the survey’s empirical results, we need to assess whether and to what extent the respondents knew about ELGA. Hoerbst et al. [18] surveyed the patients’ attitude toward ELGA in 2010. The authors found that approximately one-third of the respondents knew the term ELGA, 90 % wanted to grant their primary physician access to the health records, and 40 % stated that ELGA was an excellent idea. In a patient survey conducted at the same time of the SHARE survey, 89 % of patients indicated that the medical reports should be electronically available to both, them and the physicians treating them [32]. Thus, the Austrian health policy representatives could obtain the patients’ mandate to make the medical reports available at any time.

**Fig. 1:**
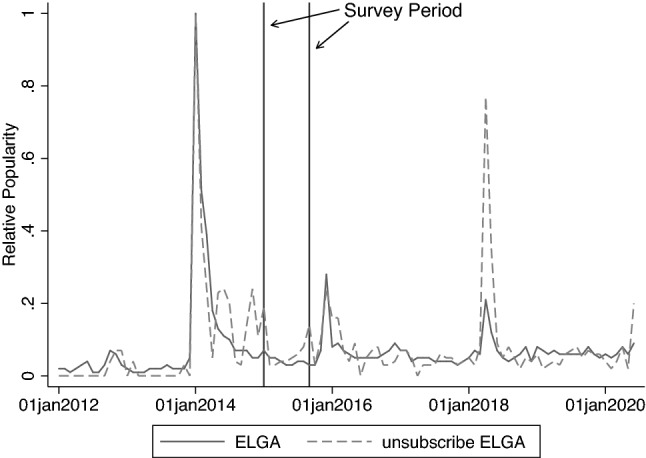
Evolution of Google Trends Data. Notes: This figure depicts the evolution of Google Trends data for the search terms “ELGA” and “unsubscribe ELGA” from 2012 to 2020. The relative popularity indicates the search interest relative to the highest point in the chart for the selected region in the specified time period

Public interest in ELGA was relatively low during the SHARE survey period. Figure 1 depicts the evolution of Google Trends data for the search terms “ELGA” and “unsubscribe ELGA” from 2012 to 2020. Public awareness was found to be particularly high at the beginning of 2014, shortly after deciding the financing bases in parliament and extensive discussions in the media. The significant increase in interest in opting out of the EHR system was fueled by the physicians’ media campaign mentioned above. Media attention and public interest slowed down in the months that followed, but was still higher than before the beginning of 2014. In particular, the discussion about unsubscribing to ELGA has resumed just before the survey period.

### Variables

We build the attitude score for ELGA following Halmdienst et al. [17]. SHARE asked the respondents whether they were aware of ELGA as well as other questions on the respondents’ attitude toward the Act. Table 1 presents the detailed parameter values. We construct a dichotomous variable (“Attitude score”) that takes the value 1 for any positive statement (“I am already using this,” “I am open to this,” “This is/would be a great help for me”) and 0 for any negative statement (“I find this daunting,” “I doubt that I would find this helpful,” “I am not interested in this,” “I do not feel comfortable around this”). The statement “I don’t know this” is kept neutral and excluded from the analysis (362 cases); this applies to cases with contradictory statements too (17 cases).

**Table 1:** Attitudes towards ELGA, weighted, multiple answers possible

Attitude	Rate selected
I don’t know this	0.11
I am already using this	0.08
I am open to this	0.37
This is/would be great help	0.04
I find this daunting	0.06
I doubt that I would find this helpful	0.05
I am not interested in this	0.31
I do not feel comfortable around this	0.04
*N*	2984

Our variables of main interest relate to health and social connectedness. The individual health (“Health general”) in SHARE is self-assessed. The health indicator is equal to 1 if the respondent stated “poor” or “fair health,” and zero otherwise. The complexity of the individual health situation, that is, whether a person has multiple contacts with the health system, represents a major advantage of an electronic health information system. We map the complexity of health status across different variables: multimorbidity (the number of diagnoses ever received), chronical illness ($1=$suffers from chronical illness), number of hospital stays in the last 12 months, and number of medication taken per week. Mental health is measured by three indicators: the EURO-D depression scale and two dummy variables equal to one for the single EURO-D dimensions “no enjoyment” and “no hopes for the future.”^2^ The other two health-related indicators cover the individual perception of the Austrian health insurance system. Respondents are asked about their satisfaction with public health insurance ($1=$satisfied), and whether they hold a supplementary private health insurance ($1=$yes).

The attitude toward ELGA can be influenced by the social connectedness of the respondents. We use the ordinal social connectedness score proposed by Litwin and Stoeckel [23] in a dichotomized form. The variable “no or small social network” is equal to 1 if the social connectedness score is smaller than 2, the social connectedness score median. Zero indicates a score of 2, 3, or 4. As indicator for digital literacy, we consider whether the person owns a tablet or smartphone. Smartphone use has been shown to be a good predictor of internet literacy of senior citizens [19].^3^

**Table 2:** Descriptive statistics

Variable	Description	*N*	Mean	S.D.	Min.	Max.
Age	Age in years (2015)	2984	69.408	9.367	51	103
Age 50–59	1 = Age category 50–59	2984	0.163		0	1
Age 60–69	1 = Age category 60–69	2984	0.361		0	1
Age 70–79	1 = Age category 70–79	2984	0.329		0	1
Age 80+	1 = Age category 80+	2984	0.148		0	1
Female	1 = Yes	2984	0.588		0	1
Partner in household	1 = Yes	2984	0.640		0	1
Has children	1 = Yes	2983	0.887		0	1
Living in a house	1 =Yes	2835	0.601		0	1
Urban area	1 = Lives in urban area	2825	0.555		0	1
Retired	1 = Retired	2948	0.740		0	1
Monthly household income p.p. in k EUR	Imputed monthly household income per person in 1000 Euro	2983$^a$	1.421	0.800	0	11.945
Higher education	1 = Obtained qualification for university entrance	2923	0.240		0	1
ELGA positive$^b$	1 = Positive attitude towards ELGA	2605	0.534		0	1
ELGA: I do not know this	1 = Selected	2967$^c$	0.122		0	1
ELGA: I am already using this	1 = Selected	2967$^c$	0.095		0	1
ELGA: I am open to this	1 = Selected	2967$^c$	0.364		0	1
ELGA: Would be a great help	1 = Selected	2967$^c$	0.037		0	1
ELGA: I find this daunting	1 = Selected	2967$^c$	0.050		0	1
ELGA: I doubt it would be helpful	1 = Selected	2967$^c$	0.049		0	1
ELGA: I am not interested	1 = Selected	2967$^c$	0.305		0	1
ELGA: I don’t feel comfortable with this	1 = Selected	2967$^c$	0.035		0	1
Owns Tablet or Smartphone	1 = Yes	2984	0.175		0	1
No or small social connectedness	1 = Social connectedness scale 0 or 1	2984	0.175		0	1
Satisfaction with public health insurance.	1 = Very or somewhat satisfied with public health insurance	2981	0.904		0	1
Has supplementary insurance	1 = Yes	2984	0.219		0	1
Poor or fair health	1 = Poor or fair self-assessed health	2984	0.338		0	1
Multimorbidity—number of diagnoses	Number of health diagnoses by a doctor	2983	1.764	1.606	0	10
Chronical illness	1 = Yes	2984	0.515		0	1
Number of hospital stays	Number of hospital stays last 12 months	2982	0.401	0.998	0	10
Number of drugs	Number of different drugs per week	2979	1.716	1.569	0	10
Depression scale EURO-D—high is depressed	Number of EURO-D symptoms	2839	2.019	2.012	0	11
Enjoyment (part of EURO-D)	1 = No enjoyment mentioned	2843	0.146		0	1
Pessimism (part of EURO-D)	1 = No hopes for the future	2843	0.070		0	1

^a^Extreme value above 30 set to missing (1 case).

^b^Final dependent variable, see variable description for construction.

^c^17 cases with contradictory statements excluded

Finally, we add the socio-economic control variables age, gender, education ($1=$higher education obtained), retirement status (retired/not retired), and average monthly household income per person in € 1,000. Moreover, we include information about the household composition: whether the partner is living in the same household, whether the person has children and lives in an urban area, and whether the respondent lives in a flat or house. Table 2 presents the descriptive statistics of the variables. In the sample, 59 % of respondents are women, the average age is 69 years, 74 % are retired, and 55.5 % live in an urban area. The average net monthly household income per person is € 1,421. 33.8 % show a poor or fair health status, while 51.5 % suffer from chronical illness. The average number of diagnoses, hospital stay in the last 12 months, and medication taken per week is 1.76, 0.4, and 1.72, respectively. As regards mental health and depression, 14.6 % mention no enjoyment, and 7 % state that they have no hopes for the future.

### Methods

Our estimation equation reads as:

1 $$\begin{aligned} \begin{aligned}&Attitude\,score_{i} = \alpha _{0} \\&\quad + \alpha _{1}\, Health\,general_{i} + \alpha _{2}\, Health\,complex_{i} \\&\quad + \alpha _{3}\, Health\,insurance_{i} \\&\quad + \alpha _{4}\, Depress_{i}+ \alpha _{5}\,Social\,connectedness_{i} \\&\quad + \alpha _{6}\, Digital_{i}+ \alpha _{7}\, SES_{i} + \epsilon _{i} \end{aligned} \end{aligned}$$

We regress the binary attitude score on independent variables of the health status, complexity of the health situation, the situation of the personal health insurance, depression indicators, social connectedness and digital literacy as well as some socio-economic control variables (SES) as described in the previous section. As the attitude score is a dichotomous variable, we use a probit estimation model, using a standard normal transformation to receive predicted values between 0 and 1. Marginal effects from a linear probability (OLS) model show fairly similar effects and are presented in the Appendix Table 6.

Our hypotheses are that a worse health status should make respondents more acceptable of ELGA, in particular that those with a complex health situation (multimorbidity, chronical illness, multiple hospital stays, number of medication, mental health variables) should be more favorable. This is the main hypothesis, because the value of ELGA—as an information system combing and checking information from different health system sources—would be maximal in the case of a complex health situation of the individual. Being satisfied with the public health insurance system might also be positively correlated with acceptance of the ELGA system. For digital literacy, we suppose that computer-literate persons per se, are also more open to digital health systems. Similarly for social connectedness: persons who are more isolated socially, might be more sceptic about modern digital health systems.

Using a cross section of the data, identification is difficult. We assume that the demographic variables are not influenced by attitudes towards ELGA, as they are mostly pre-determined. We would also argue that actual health is not impacted by these attitudes. On the other hand, we cannot exclude that our measure of computer-literacy is influenced by attitudes towards digital health; there is some room for reverse causation here.

## Results

**Table 3:** Main results: Marginal effects from probit estimation with positive attitude towards ELGA as binary dependent variable

	(1)	(2)	(3)	(4)	(5)	(6)	(7)	(8)
Poor or fair health	0.014	– 0.034	– 0.032	0.002	– 0.017	0.014	0.014	0.022
	(0.030)	(0.033)	(0.033)	(0.031)	(0.033)	(0.033)	(0.030)	(0.031)
Multimorbidity—number of diagnoses		0.039***						
		(0.009)						
Chronical illness			0.087***					
			(0.030)					
Number of hospital stays				0.027**				
				(0.013)				
Number of drugs					0.026***			
					(0.010)			
Depression scale EURO-D—high is depressed						– 0.000		
						(0.008)		
No enjoyment (part of EURO-D)							– 0.083**	
							(0.041)	
Pessimism (part of EURO-D)								– 0.131**
								(0.059)
Satisfaction with public health insurance	0.005	0.012	0.006	0.004	0.005	0.007	0.006	0.007
	(0.047)	(0.048)	(0.048)	(0.047)	(0.048)	(0.049)	(0.049)	(0.049)
Has supplementary insurance	0.036	0.035	0.031	0.031	0.034	0.035	0.034	0.037
	(0.034)	(0.034)	(0.034)	(0.034)	(0.034)	(0.034)	(0.034)	(0.034)
Owns tablet or smartphone	0.145***	0.142***	0.146***	0.141***	0.142***	0.138***	0.141***	0.143***
	(0.038)	(0.038)	(0.038)	(0.038)	(0.038)	(0.038)	(0.038)	(0.038)
No or small social connectedness	– 0.109***	– 0.103***	– 0.108***	– 0.107***	– 0.107***	– 0.103***	– 0.104***	– 0.098***
	(0.037)	(0.037)	(0.036)	(0.037)	(0.037)	(0.037)	(0.037)	(0.037)
Age	– 0.002	– 0.004**	– 0.002	– 0.003	– 0.003*	– 0.002	– 0.002	– 0.002
	(0.002)	(0.002)	(0.002)	(0.002)	(0.002)	(0.002)	(0.002)	(0.002)
Female	0.023	0.020	0.021	0.022	0.021	0.027	0.027	0.027
	(0.029)	(0.029)	(0.029)	(0.029)	(0.029)	(0.030)	(0.029)	(0.029)
Partner in household	0.051	0.048	0.050	0.051	0.049	0.047	0.046	0.044
	(0.032)	(0.032)	(0.032)	(0.032)	(0.032)	(0.032)	(0.032)	(0.032)
Has children	– 0.051	– 0.041	– 0.051	– 0.049	– 0.046	– 0.049	– 0.048	– 0.052
	(0.044)	(0.045)	(0.044)	(0.044)	(0.044)	(0.045)	(0.044)	(0.045)
Living in a house	0.076**	0.079**	0.079**	0.077**	0.083**	0.074**	0.074**	0.072**
	(0.033)	(0.034)	(0.033)	(0.033)	(0.033)	(0.033)	(0.033)	(0.033)
Urban area	0.067**	0.056	0.068**	0.068**	0.062*	0.075**	0.073**	0.074**
	(0.034)	(0.034)	(0.034)	(0.034)	(0.034)	(0.034)	(0.033)	(0.034)
Retired	0.023	0.018	0.020	0.022	0.018	0.016	0.014	0.018
	(0.034)	(0.034)	(0.034)	(0.034)	(0.034)	(0.035)	(0.034)	(0.035)
Monthly household income p.p. in k EUR	0.055***	0.055***	0.056***	0.054***	0.057***	0.053***	0.052**	0.050**
	(0.020)	(0.020)	(0.020)	(0.020)	(0.020)	(0.020)	(0.020)	(0.020)
Higher education	0.037	0.042	0.038	0.037	0.043	0.034	0.036	0.034
	(0.035)	(0.035)	(0.035)	(0.035)	(0.035)	(0.036)	(0.036)	(0.036)
*N*	2382	2381	2382	2382	2378	2298	2300	2300

Standard errors in parentheses. *$p<0.10$, ** $p<0.05$, *** $p<0.01$

Table 3 presents the main results using the SHARE probability weights. Appendix Table 7 shows the corresponding unweighted results, which are basically unchanged. We report the marginal effects from the underlying probit estimates for positive attitude toward ELGA. Column (1) depicts the main specification of personal characteristics and general health status. We enrich this specification with additional variables for contact diversity within the health system in columns (2) to (5), and information on mental health in columns (6) to (8).

Self-assessed health, in general, is not associated with a positive or negative attitude toward ELGA. In contrast, the respondents would be more positive toward ELGA if their health conditions are complex. If they suffer from multimorbidity or a chronical illness, regularly take more medication, and spent more time in hospital in the previous year, they would be more willing to accept EHR. The effects are sizable, with a 4 percentage points (pp) increase in positive attitude for multimorbidity (the number of diagnoses), 9 pp for chronical illness, and around 3 pp each for the number of hospital stays and drugs. These respondents seem to realize the main advantages of EHR, namely, the coordination between and information about different health care providers.

Column (6) gives the EURO-D depression scale as an indicator for mental health. In general, depression is not correlated with the attitude toward ELGA. However, two sub-items of depression scale are negatively associated with the ELGA attitude. “Having no enjoyment” and “being pessimistic” reduce the probability of a positive attitude toward ELGA by 8.3 and 13.1 pp, respectively. Satisfaction with public health insurance and holding a supplementary private health insurance are not associated with the attitude toward ELGA.

The largest association with ELGA can be observed for previous disposition toward new technologies and social connectedness. Elderly persons owning a tablet or smartphone are 14.5 % more likely to have a positive attitude toward ELGA. This is consistent with previous evidence that familiarity with IT leads to a better understanding of functions and usefulness of health IT [29]. Likewise, endowment of an elderly person with no or only a small social network is associated with a 11 pp lower acceptance of ELGA; this confirms a finding for Italian hospitals [26].

The results for the other control variables do not change across the table columns; that is, the impact of personal socio-economic characteristics is very stable. We find a positive and significant impact of “living in a house,” “living in urban area,” and “monthly household income per person.” The marginal effects of these variables are between 5 and 8.3 pp, compared to the average of 53.4 % for a positive attitude toward ELGA. Household income, living in a house and living in an urban area stand for an urban, higher-educated lifestyle which has been found to be positively related to trust in science [15]. Most variables, such as age, being female, being retired, having children, and education, remain insignificant.^4^

**Table 4:** Results by gender: Marginal effects from probit estimation with positive attitude towards ELGA as binary dependent variable

	(1)	(2)	(3)	(4)	(5)	(6)	(7)	(8)
Panel A: Females								
Poor or fair health	− 0.024	− 0.080*	− 0.062	− 0.039	− 0.058	− 0.020	− 0.020	− 0.009
	(0.038)	(0.041)	(0.042)	(0.039)	(0.041)	(0.041)	(0.039)	(0.039)
Multimorbidity—number of diagnoses		0.048***						
		(0.012)						
Chronical illness			0.078**					
			(0.038)					
Number of hospital stays				0.035**				
				(0.016)				
Number of drugs					0.029**			
					(0.013)			
Depression scale EURO-D—high is depressed						− 0.000		
						(0.009)		
No enjoyment (part of EURO-D)							− 0.113**	
							(0.048)	
Pessimism (part of EURO-D)								− 0.147*
								(0.080)
No or small social connectedness	− 0.122**	− 0.118**	− 0.123**	− 0.121**	− 0.121**	− 0.121**	− 0.119**	− 0.114**
	(0.052)	(0.053)	(0.052)	(0.052)	(0.052)	(0.052)	(0.052)	(0.053)
Number of observations	1379	1378	1379	1379	1377	1343	1343	1343
Panel B: Males								
Poor or fair health	0.073	0.041	0.019	0.067	0.051	0.067	0.069	0.072
	(0.048)	(0.054)	(0.053)	(0.050)	(0.053)	(0.055)	(0.048)	(0.048)
Multimorbidity—number of diagnoses		0.023						
		(0.015)						
Chronical illness			0.092*					
			(0.047)					
Number of hospital stays				0.012				
				(0.025)				
Number of drugs					0.018			
					(0.015)			
Depression scale EURO-D—high is depressed						0.001		
						(0.014)		
No enjoyment (part of EURO-D)							− 0.042	
							(0.068)	
Pessimism (part of EURO-D)								− 0.083
								(0.091)
No or small social connectedness	− 0.111**	− 0.106**	− 0.110**	− 0.110**	− 0.108**	− 0.101**	− 0.102**	− 0.098*
	(0.051)	(0.051)	(0.051)	(0.051)	(0.051)	(0.051)	(0.051)	(0.051)
Number of observations	1003	1003	1003	1003	1001	955	957	957

Standard errors in parentheses. * $p<0.10$, ** $p<0.05$, *** $p<0.01$

Table 4 shows the results for males and females separately for the variables of main interest (health and social connectedness). Females react much more strongly to the complexity of health: all indicators associated with health complexity are positive and highly significant for them. For men, the impact of complexity variables is basically zero; only the indicator of chronical illness is positively correlated at the 10 % significance level, with a positive attitude toward ELGA. The same holds true for mental health conditions. Females who cannot find enjoyment in life or are pessimistic express a significantly less positive attitude toward ELGA (11.3 and 14.7 pp, respectively). For men, we do not find statistically significant coefficients. It seems that in the case of men, health considerations do not change attitudes toward an electronic health record which is similar to attitudes towards other health-related uses of smart-phones [17]. Social connectedness plays an important role for both sexes, but the (negative) coefficients are slightly higher for females.

**Table 5:** Results by age: Marginal effects from probit estimation with positive attitude towards ELGA as binary dependent variable

	(1)	(2)	(3)	(4)	(5)	(6)	(7)	(8)
Panel A: Age 50–69								
Poor or fair health	0.016	− 0.016	− 0.044	0.003	− 0.019	0.023	0.015	0.026
	(0.044)	(0.048)	(0.047)	(0.045)	(0.048)	(0.047)	(0.044)	(0.044)
Multimorbidity—number of diagnoses		0.031**						
		(0.015)						
Chronical illness			0.114***					
			(0.040)					
Number of hospital stays				0.036*				
				(0.021)				
Number of drugs					0.032**			
					(0.016)			
Depression scale EURO-D—high is depressed						− 0.006		
						(0.011)		
No enjoyment (part of EURO-D)							− 0.141**	
							(0.057)	
Pessimism (part of EURO-D)								− 0.220**
								(0.090)
No or small social connectedness	− 0.098**	− 0.091*	− 0.099**	− 0.095*	− 0.095*	− 0.092*	− 0.091*	− 0.080
	(0.049)	(0.049)	(0.049)	(0.049)	(0.049)	(0.049)	(0.050)	(0.050)
Number of observations	1323	1322	1323	1323	1322	1285	1287	1287
Panel B: Age 70+								
Poor or fair health	0.009	− 0.065	− 0.010	− 0.004	− 0.015	− 0.015	0.010	0.011
	(0.035)	(0.039)	(0.042)	(0.036)	(0.039)	(0.040)	(0.036)	(0.036)
Multimorbidity—number of diagnoses		0.049***						
		(0.011)						
Chronical illness			0.036					
			(0.040)					
Number of hospital stays				0.021				
				(0.017)				
Number of drugs					0.020*			
					(0.012)			
Depression scale EURO-D—high is depressed						0.014		
						(0.010)		
No enjoyment (part of EURO-D)							0.019	
							(0.050)	
Pessimism (part of EURO-D)								− 0.010
								(0.065)
No or small social connectedness	− 0.137***	− 0.133***	− 0.135***	− 0.136***	− 0.136***	− 0.135***	− 0.137***	− 0.137***
	(0.047)	(0.047)	(0.047)	(0.047)	(0.047)	(0.048)	(0.048)	(0.048)
Number of observations	1059	1059	1059	1059	1056	1013	1013	1013

Standard errors in parentheses. * $p<0.10$, ** $p<0.05$, *** $p<0.01$

Table 5 splits our sample by age. Panel A presents the results for respondents aged 50–69, and Panel B shows the results for persons beyond age 69. The estimation results indicate that patients in the older age group are less convinced (or informed) about the usefulness of ELGA. The coefficients of health complexity variables are insignificant, with the exception of multimorbidity. In contrast, younger respondents react much more strongly to the indicators of health complexity. Note that persons below age 70 with a pessimistic life attitude are hardly interested in EHR. Their probability of positive attitude toward ELGA falls by 22 pp, representing the highest point estimate (in absolute values) for all coefficients. Social integration plays a more important role for elderly persons: lack of social integration reduces the positive attitude toward ELGA by 9.8 pp for persons aged 50–69 and by 13.7 pp for persons aged above 69. Well-connected elderly persons seem to learn from peers the advantages of EHR and thus welcome them.

## Conclusions and discussion

In this study, we examined how personal health and the complexities thereof affect the willingness of elderly Austrians to adopt EHR. Using data from the sixth wave of the SHARE survey in Austria, we find that, apart from the inclination to adopt new technologies, the complexity of individual health problems and social integration of individuals influence the acceptance of EHR. The higher the degree of multimorbidity, the more medication is prescribed, and the higher the number of hospital admissions, the higher is the acceptance of EHR. Chronic illness also has a positive effect on EHR acceptance, whereas a pessimistic attitude and lack of joy in life as indicators of depressive mood have a negative impact. The results are mainly based on females and the younger patients aged between 50 and 70. People poorly connected socially indicate lower acceptance of EHR. This applies to both sexes and all age groups, but is particularly pronounced for people above 70.

*Discussion:*  The main result of our study that the degree of multimorbidity and the intensity of health care service use increase the acceptance of EHR is confirmed in the recent patient survey conducted by ELGA GmbH [12]. There, the heavy users are identified as clear ELGA supporters. They are frequently ill, go to the doctor often and use the health care system intensively. ELGA is of great benefit to this group, because the patients always have their medical findings and their list of medications at hand.

The second group for which the ELGA GmbH [12] evaluation also shows high EHR acceptance are patients with increasing awareness of prevention and health promotion. They take care of their health and still go to the doctor frequently. Given their average age of under 50, they are obviously underrepresented in our study of older people.

The novel contribution of our study—not least in comparison to the ELGA GmbH [12] evaluation study cited above—is as follows:

- The 2020 ELGA evaluation study is based on a survey of the Austrian population between 16 and 75 years. It uses about 1,300 predominantly computer-assisted web interviews. The sample of our analysis includes 3,000 face-to-face interviews in the 50+ age group. For this group, the provision of health care services and the availability of their documentation are particularly important.
- While the available evaluation studies use simple descriptive analyses, our regression approach allows ceteris paribus statements by simultaneously controlling for potential determinants of EHR acceptance. In addition, the SHARE data include important variables on socio-economic status or individual life circumstances that are not asked in the ELGA evaluation studies.
- Another key strength of the SHARE survey is that the paper-and-pencil questionnaire on attitudes toward new technologies was completed by more than 90 % of respondents (see page 5). The high response rate indicates that potential selection effects are of minor significance.

*Conclusions:*  Although our analysis does not attempt a strict causal interpretation of the associations, we can draw important conclusions. We need more intervention to enhance the health literacy of older people and help patients better understand the complexity of health problems and the role that information plays in managing them. This can be done through targeted campaigns by health insurance funds as well as better educational efforts by doctors and other health care providers. Furthermore, we find that societal efforts to provide older people with access to information technologies and electronic media can both make their daily lives easier and significantly increase their acceptance and adoption of EHR and other platforms collecting individual data in compliance with comprehensive data protection principles.

Finally, the results clearly show that optimism and enjoyment of life as well as high degree of social connectedness are important determinants of trust in EHR. All measures to strengthen the mental health of older people and promote their social integration obviously generate spillovers and make them more willing to provide access to their health data regardless of location. The importance of this access from a public health perspective was illustrated in Austria with regard to the COVID-19 pandemic when it organized both the distribution of free self-tests and the national COVID vaccination registry via the ELGA platform.

## Appendix

See Tables 6 and 7.

**Table 6:** Marginal effects from OLS estimation with positive attitude towards ELGA as binary dependent variable

	(1)	(2)	(3)	(4)	(5)	(6)	(7)	(8)
Poor or fair health	0.014	− 0.033	− 0.030	0.002	− 0.016	0.013	0.014	0.021
	(0.029)	(0.032)	(0.032)	(0.030)	(0.032)	(0.032)	(0.029)	(0.030)
Multimorbidity—number diagnoses		0.037***						
		(0.009)						
Chronical illness			0.084***					
			(0.028)					
Number of hospital stays				0.025**				
				(0.012)				
Number of drugs					0.026***			
					(0.010)			
Depression scale EURO-D—high is depressed						− 0.000		
						(0.008)		
No enjoyment (part of EURO-D)							− 0.081**	
							(0.039)	
Pessimism (part of EURO-D)								− 0.123**
								(0.055)
Satisfaction with public health insurance	0.006	0.012	0.006	0.005	0.006	0.007	0.006	0.007
	(0.045)	(0.046)	(0.046)	(0.045)	(0.046)	(0.047)	(0.047)	(0.047)
Has supplementary insurance	0.033	0.032	0.028	0.028	0.031	0.032	0.031	0.033
	(0.032)	(0.032)	(0.032)	(0.032)	(0.032)	(0.032)	(0.032)	(0.032)
Owns tablet or smartphone	0.137***	0.133***	0.137***	0.134***	0.134***	0.131***	0.133***	0.135***
	(0.035)	(0.035)	(0.035)	(0.035)	(0.035)	(0.035)	(0.035)	(0.035)
No or small social connectedness	− 0.106***	− 0.099***	− 0.104***	− 0.103***	− 0.103***	− 0.100***	− 0.101***	− 0.095***
	(0.035)	(0.035)	(0.035)	(0.035)	(0.035)	(0.035)	(0.036)	(0.036)
Age in years (2015)	− 0.002	− 0.004**	− 0.002	− 0.002	− 0.003*	− 0.002	− 0.002	− 0.002
	(0.002)	(0.002)	(0.002)	(0.002)	(0.002)	(0.002)	(0.002)	(0.002)
Female	0.023	0.019	0.020	0.021	0.020	0.026	0.027	0.026
	(0.028)	(0.028)	(0.027)	(0.028)	(0.028)	(0.029)	(0.028)	(0.028)
Partner in household	0.049	0.046	0.049	0.050	0.047	0.045	0.044	0.042
	(0.031)	(0.030)	(0.031)	(0.031)	(0.031)	(0.031)	(0.031)	(0.031)
Has children	− 0.048	− 0.038	− 0.047	− 0.045	− 0.043	− 0.046	− 0.045	− 0.050
	(0.042)	(0.042)	(0.042)	(0.042)	(0.042)	(0.043)	(0.042)	(0.043)
Living in a house	0.073**	0.076**	0.076**	0.074**	0.079**	0.071**	0.071**	0.069**
	(0.032)	(0.032)	(0.031)	(0.032)	(0.032)	(0.032)	(0.032)	(0.032)
Urban area	0.065**	0.053*	0.066**	0.065**	0.059*	0.072**	0.070**	0.070**
	(0.032)	(0.032)	(0.032)	(0.032)	(0.032)	(0.032)	(0.032)	(0.032)
Retired	0.022	0.016	0.018	0.020	0.017	0.015	0.013	0.017
	(0.033)	(0.033)	(0.033)	(0.033)	(0.033)	(0.033)	(0.033)	(0.033)
Monthly household income p.p. in k EUR	0.052***	0.053***	0.054***	0.051***	0.054***	0.051***	0.050***	0.048**
	(0.019)	(0.019)	(0.019)	(0.019)	(0.019)	(0.019)	(0.019)	(0.019)
Higher education	0.035	0.040	0.036	0.035	0.040	0.032	0.035	0.032
	(0.034)	(0.034)	(0.034)	(0.034)	(0.034)	(0.034)	(0.034)	(0.035)
Constant	0.474***	0.513***	0.458***	0.483***	0.494***	0.451***	0.449***	0.462***
	(0.123)	(0.123)	(0.124)	(0.123)	(0.124)	(0.125)	(0.125)	(0.124)
N	2382	2381	2382	2382	2378	2298	2300	2300

Standard errors in parentheses. * $p<0.10$, ** $p<0.05$, *** $p<0.01$

**Table 7:** Results (unweighted): Marginal effects from probit estimation with positive attitude towards ELGA as binary dependent variable

	(1)	(2)	(3)	(4)	(5)	(6)	(7)	(8)
Poor or fair health	− 0.009	− 0.060**	− 0.029	− 0.021	− 0.042*	− 0.011	− 0.006	− 0.002
	(0.023)	(0.025)	(0.026)	(0.024)	(0.025)	(0.026)	(0.024)	(0.024)
Multimorbidity—number of diagnoses		0.038***						
		(0.008)						
Chronical illness			0.038					
			(0.024)					
Number of hospital stays				0.025**				
				(0.011)				
Number of drugs					0.027***			
					(0.008)			
Depression scale EURO-D—high is depressed						0.003		
						(0.006)		
No enjoyment (part of EURO-D)							− 0.054*	
							(0.031)	
Pessimism (part of EURO-D)								− 0.062
								(0.046)
Satisfaction with public health insurance	0.076**	0.088**	0.079**	0.075**	0.079**	0.078**	0.077**	0.076**
	(0.035)	(0.035)	(0.035)	(0.035)	(0.035)	(0.036)	(0.036)	(0.036)
Has supplementary insurance	0.001	0.000	− 0.001	− 0.004	− 0.000	0.000	0.001	0.002
	(0.026)	(0.026)	(0.026)	(0.026)	(0.026)	(0.027)	(0.027)	(0.027)
Owns tablet or smartphone	0.199***	0.199***	0.199***	0.195***	0.197***	0.195***	0.196***	0.196***
	(0.028)	(0.028)	(0.028)	(0.028)	(0.028)	(0.029)	(0.029)	(0.029)
No or small social connectedness	− 0.107***	− 0.100***	− 0.105***	− 0.105***	− 0.103***	− 0.103***	− 0.103***	− 0.102***
	(0.028)	(0.029)	(0.028)	(0.028)	(0.029)	(0.029)	(0.029)	(0.029)
Age	− 0.001	− 0.002	− 0.001	− 0.001	− 0.002	− 0.000	− 0.000	− 0.000
	(0.001)	(0.001)	(0.001)	(0.001)	(0.001)	(0.001)	(0.001)	(0.001)
Female	0.027	0.025	0.027	0.026	0.026	0.024	0.027	0.026
	(0.022)	(0.022)	(0.022)	(0.022)	(0.022)	(0.023)	(0.023)	(0.023)
Partner in household	0.077***	0.074***	0.078***	0.077***	0.076***	0.073***	0.073***	0.071***
	(0.024)	(0.024)	(0.024)	(0.024)	(0.024)	(0.025)	(0.025)	(0.025)
Has children	− 0.034	− 0.025	− 0.035	− 0.033	− 0.031	− 0.034	− 0.035	− 0.034
	(0.036)	(0.036)	(0.036)	(0.036)	(0.036)	(0.036)	(0.036)	(0.036)
Living in a house	0.065**	0.069***	0.066**	0.064**	0.071***	0.061**	0.061**	0.060**
	(0.026)	(0.026)	(0.026)	(0.026)	(0.026)	(0.026)	(0.026)	(0.026)
Urban area	0.066**	0.059**	0.066***	0.066**	0.062**	0.068***	0.066**	0.068***
	(0.025)	(0.026)	(0.025)	(0.025)	(0.026)	(0.026)	(0.026)	(0.026)
Retired	0.004	0.001	0.003	0.004	0.001	0.001	0.001	0.001
	(0.027)	(0.027)	(0.027)	(0.027)	(0.027)	(0.027)	(0.027)	(0.027)
Monthly household income p.p. in k EUR	0.058***	0.059***	0.058***	0.057***	0.059***	0.055***	0.055***	0.054***
	(0.014)	(0.014)	(0.014)	(0.014)	(0.014)	(0.015)	(0.015)	(0.015)
Higher education	0.050*	0.051*	0.049*	0.050*	0.053**	0.046*	0.048*	0.047*
	(0.026)	(0.027)	(0.026)	(0.026)	(0.027)	(0.027)	(0.027)	(0.027)
*N*	2382	2381	2382	2382	2378	2298	2300	2300

Standard errors in parentheses. * $p<0.10$, ** $p<0.05$, *** $p<0.01$

## Footnotes

1 Leisch and Sabutsch [22] document the historical development of ELGA in detail. The authors respond to criticism, present the improvements realized in practical handling and provide an outlook on future extensions.

2 The other single EURO-D dimensions give insignificant results.

3 For positive correlations between smartphone use and technology attitudes, see also Kör et al. [21].

4 Education is positively correlated with income. If income is excluded, we find a positive and significant association between education and acceptance of ELGA.
